# Supplementary material for: Chromothripsis during telomere crisis is independent of NHEJ, and consistent with a replicative origin
Source: Genome Res. 2019 May;29(5):737–49. doi: 10.1101/gr.240705.118 (PMC6499312; doi:10.1101/gr.240705.118)
Supplement: Supplemental Material [file supp_gr.240705.118_Supplemental_file_1.zip › contigs/annotated_contigs/DB53/contig.2.DB53_length_701_mean_cov_6.57774607703.docx]

**DB53_length_701_mean_cov_6.57774607703**

```
TAAGGGCTGAGTAGGGTATTATTCATAATTGTTATTTAATTTGGTCTTCTTCCCATCTTCCTTCTAACAGAAGGCTTTTCTGCCTTGAA
 >chr8:129902915-129903287 - E=5e-212
AAATTACACCATTTAAAGATGCCCTATGTCTGTGATTATATGCTATATAACCTGGCAAATATTCTTCAAATAAAAGCCCACAAGTCCAG

TAAAATTTGCAGTAGCAGATGACATTTTCACCAAAGAACAGAGCCAGTTTTTCACGAAGGTAGAAGAAGGACCCACCCCCACTTGACTA

TCTTGTTTATTTGACTGACTTCATTAATCCCTGGAGCTTCCTGATAACTCCAGGAAAGCATCCAAACTCATCTCTGTCTGGCTCCTTCT

TGTGTCACCAGCCCC|A|AGAACTGAAGTACTGTCAAGAACTTTCTTTTTGTGAGCAGAACTTTACATTCCTAAGATAATTTTTAAAAA
                >chr8:121496804-121497134 + E=9e-184
TACAGTTTTATTGAGATATAATGCATACCATACATTTTACCCTTTTAAAGTTTACAATTCAGTGGCTTTTAAAATATAATCACAGAATT

GTGCAACTATCACCACAGTCAAGTTTAGGGCATCTTAATCTCCCCGGAAAGAAACCCCATACGCGTTAGCAATCATTGCGCATTTCCAC

CACCCATTCCTCCTCCCTACCCCTAAGCAATCTATTTTGGTCTGTATAGATTTGACTGTTCTGGACATGTCATGGAAGTG
```
